# Two for the price of one: Concurrent learning of words and phonotactic regularities from continuous speech

**DOI:** 10.1371/journal.pone.0253039

**Published:** 2021-06-11

**Authors:** Viridiana L. Benitez, Jenny R. Saffran

**Affiliations:** 1 Department of Psychology, Arizona State University, Tempe, AZ, United States of America; 2 Waisman Center, University of Wisconsin-Madison, Madison, WI, United States of America; Abertay University, UNITED KINGDOM

## Abstract

To acquire the words of their language, learners face the challenge of tracking regularities at multiple levels of abstraction from continuous speech. In the current study, we examined adults’ ability to track two types of regularities from a continuous artificial speech stream: the individual words in the speech stream (token level information), and a phonotactic pattern shared by a subset of those words (type level information). We additionally manipulated exposure time to the language to examine the relationship between the acquisition of these two regularities. Using a ratings test procedure, we found that adults can extract both the words in the language and their phonotactic patterns from continuous speech in as little as 3.5 minutes of listening time. Results from a 2AFC testing method provide converging evidence that adults rapidly learn both words and their phonotactic patterns. Together, the findings suggest that adults are capable of concurrently tracking regularities at multiple levels of abstraction from brief exposures to a continuous stream of speech.

## Introduction

One of the first steps in language learning is identifying how sounds combine to form words. This task requires learners to be sensitive to regularities at multiple levels. Language learners must track regularities at the token level, such as the individual lexical items present in the speech stream. Additionally, learners must track patterns at the type level, such as the phonological regularities shared *among* those lexical items. How do learners navigate acquiring both specific (token) and generalizable (type) patterns from continuous speech?

### Learning from continuous speech

Previous research has demonstrated that learners are sensitive to a wide array of cues that aid in parsing the speech stream into individual word units (for reviews, see [1–4]). One type of cue that learners use to segment speech are co-occurrence statistics like transitional probabilities, which are higher (on average) within words than between words. After a few minutes of exposure to a continuous stream of speech, where transitional probabilities are the only signal to word boundaries, both infants and adults can discriminate combinations of syllables that reliably co-occurred in the stream from those that were less likely to co-occur [5–7]. By tracking syllable-based statistical regularities, learners can break into fluent speech and begin to identify the individual word units.

In addition to discovering word tokens in continuous speech, learners must also track type-level (generalizable) information about those words, such as phonotactic regularities, which specify the positional restrictions on phoneme sequences in a language. These patterns are complex: they are language specific, probabilistic, and often contain multiple features. These multiple features may be adjacent, where two or more phonemes consistently occur one right after the other, or nonadjacent, where other phonemes appear between phonemes that consistently occur together. An example of an adjacent phonotactic pattern is the sequence /fr/, which in English occurs most often at the beginning of words, and occasionally in the middle of words (e.g., *frog*, *afraid*), but never at the ends of words. An example of a nonadjacent phonotactic pattern is the consonant root /k/-/t/-/b/ in Hebrew, with a general meaning of “to write”, where the consonants occur together in a specific order with variation in the intervening vowels (e.g., *katab* which means “he wrote”, *koteb* which means “writer”).

Typically developing infants show knowledge concerning both adjacent and nonadjacent phonotactic regularities in their native language by 10 months of age, suggesting they are able to extract them from the speech that they hear [8–12]. Lab-learning studies also demonstrate that learners can identify novel phonotactic patterns shared among words in a continuous stream of speech, generalizing the patterns beyond the words presented in the speech stream [13–15]. When exposed to a continuous stream of speech where transitional probabilities signaled the word boundaries, infants were able to generalize a novel phonotactic regularity consisting of one sound feature constrained to the onset of words (/t/ occurs at the beginning of words; [15]). In a language containing nonadjacent phonotactic regularities, adults were able to abstract nonadjacent consonantal patterns with intervening vowels (e.g., /p/-/d/-/k/) from a continuous stream of speech, generalizing that pattern to novel items not presented in the speech stream [13]. These findings show that learners can track regularities at the type level, identifying the set of phonemes that most likely occur in the words present in a fluent stream of speech.

Although it is clear that learners can extract the individual words and the phonotactic patterns of a continuous stream of speech, it is unclear whether learners are able to track both types of patterns from the same set of input. Specifically, tracking both the individual words in the language and the features that are consistent across those words requires learners to track patterns at different levels of abstraction. Learners must be able to identify token information–the individual words that compose the speech stream, as well as type information–the patterns that are present across those words and that generalize to novel words in the same language, from the same speech stream. To date, no study has tested how individuals learn both types and tokens from the same set of input. For example, although Sahni et al. [15] demonstrated that infant learners can detect a phonotactic pattern in a continuous stream of speech, infants were only tested on novel generalization items that followed the phonotactic pattern, leaving it unclear whether or not infants also discovered the individual words in the language. Likewise, although Adriaans & Kager [13] demonstrated that adults learn phonotactic regularities from a continuous stream of speech, their speech stream contained words that only occurred once (without repetitions), so it is not clear if adults segmented and learned the words in addition to their phonotactic patterns. These questions are important because learners demonstrate knowledge of some words in their native language as well as the phonotactic patterns that are shared by those words by their first year of life [8–12,16–18], yet current proposals differ with respect to how learners may be able to acquire each type of pattern from continuous speech [4,19]. When learners are faced with a novel speech stream, how do they navigate acquiring both the individual words and the phonotactic patterns of that speech stream? Do learners extract both patterns concurrently or do they track one before learning the other? Is there a preference for one type of regularity over the other?

### Acquiring regularities at multiple levels of abstraction

The question of learning patterns at multiple levels of abstraction has been the focus of a limited set of studies examining the learning of statistical regularities in the visual domain [20–22] as well as a larger set of studies when considering extracting regularities from speech [4,14,23–30]. When examining the learning of statistical regularities in the visual domain, adults are successful at tracking regularities at both the token level and the type level [20,21]. In a study presenting adults with sequences of scenes, adults tracked the statistics of which individual scenes were likely to follow each other (a specific kitchen scene was followed by a specific forest scene), and which categories of scenes were likely to follow each other (kitchen scenes were likely to be followed by forest scenes; [20]). Further, when presented with statistical regularities about sequences of objects at both the item level and category level simultaneously, learners seem to have a preference, focusing on tracking item-level regularities rather than category level regularities [21]. These studies demonstrate that although learners are sensitive to regularities at multiple levels of abstraction in the visual domain, they may prefer tracking regularities at the individual token level over the type level.

In the speech domain, numerous studies have demonstrated that learners can abstract a rule from specific speech input and generalize that rule to novel stimuli–that is, acquiring a type-level abstraction [23–30]. However, only a limited set of studies have examined the ability to extract words (tokens) and learn rules (types) from the same continuous stream of speech, albeit tested across different learners. Results from these studies suggest that abstracting a rule from a continuous stream of speech containing transitional probability information may be difficult [23,27,30], but see [13]. Peña et al. [27] examined whether adult learners were capable of using non-adjacent transitional probabilities between syllables composing tri-syllabic words (in the item AxB, syllable A always predicted syllable B, and syllable x varied between one of three syllables) to segment a continuous speech stream. In a separate experiment, a new group of adults were presented with the same speech stream to examine if learners were able to track the abstract rule composing those words (if learners generalize the “rule” structuring all AxB items to identify AyB as valid, with y being a syllable not present in the speech stream). They found adults were only able to track the non-adjacent probabilities to identify the words in the continuous stream of speech, but they did not abstract the rule about the words in the speech stream unless segmentation cues (a short pause between items) were added [27]. Peña et al. [27]’s findings suggest adults’ tracking of individual item level information may compete with adults’ tracking of rule-level information when presented with a continuous stream of speech.

In sum, although previous research has indicated that learners can abstract rules beyond the speech to which they are exposed, findings from both the visual and speech domain examining learning of item-level information and type-level information suggest that this information may compete. When it comes to learning both the words and their phonotactics from continuous speech, learners may therefore find it difficult to abstract both types of patterns from the same continuous speech stream.

### Adjacent and nonadjacent dependency learning

Languages contain phonotactic regularities that are composed of adjacent elements and/or nonadjacent elements [9]. The case of nonadjacent phonotactic patterns poses an additional challenge for learners’ ability to track these patterns in unsegmented speech, as it requires learners to track differing distributional structures: words composed of adjacent dependencies and phonotactic patterns composed of nonadjacent dependencies. Previous research has demonstrated that under certain conditions, infants and adult learners are successful at tracking novel nonadjacent dependencies from artificial languages [31–33]. For example, when presented with a continuous stream of speech, adults failed at tracking nonadjacent dependencies between syllables, but succeeded at tracking nonadjacent dependencies between consonants or vowels [33]. However, only two studies have examined concurrent learning of adjacent and nonadjacent dependencies in the same learners [34,35]. When adults were presented with an artificial grammar (phrases containing segmented novel words) containing both adjacent and nonadjacent elements, adults were able to learn both types of structures [34]. Consistent with this finding, Vuong et al. [35] measured online learning of adjacent and nonadjacent dependencies using a serial reaction time task, finding that individual learners acquired both types of regularities.

Together, these studies suggest learners should be able track adjacent patterns to find words and nonadjacent patterns to identify the phonotactics of continuous speech. However, this is currently an open question; no study has examined the learning of words (via adjacent regularities) and a phonotactic pattern (via non-adjacent regularities) from a continuous stream of speech.

### Mutually dependent regularities

If learners are able to track both types of patterns from the same continuous stream of speech, an additional question is if they track one regularity before the other. Infants demonstrate some knowledge of the words of their native language as young as 6 months of age [16], and have the ability to segment a novel, continuous stream of speech into its individual word units by 8 months of age [5,6]. By 10 months of age, infants also demonstrate knowledge of the phonotactic regularities of their native language [8–12], and by this age, also show rapid acquisition of novel phonotactic regularities from artificial speech materials [15,36–42]. Further, there is evidence that one regularity supports the learning of the other regularity. Learners use phonological regularities to segment the speech stream into its individual word units [43–47]. Additionally, exposure to clearly-segmented individual words allows learners to extract the phonotactic regularities of those words [36–40]. Thus, learners may need one pattern in order to identify the other.

One possibility is that in order to identify the phonotactic patterns shared among words, learners must first identify the individual words in the language [4,48]. Exposure to clearly segmented individual words allows learners to rapidly track their phonotactic patterns. For example, when presented with lists of isolated words that adhere to novel sound constraints (e.g., /b/ only occurs at the beginning of a word, and /p/ only at the end), infants and adults can rapidly acquire the phonotactic regularities that characterize those words [37,38,40,49]. If learners first identify where words begin and end to discover candidate words, the phonotactic regularities shared amongst those words can be discerned. From this perspective, tracking phonotactic patterns from continuous speech may be dependent on first finding and representing the individual lexical items in the speech stream.

A separate literature documents the important role of phonological regularities in segmenting speech into its component words. Once learners have acquired the phonological properties that constrain the words of their native language, they can use those patterns to parse the speech stream into its component words [43–45], sometimes preferring native-language phonological patterns over transitional probability information to segment speech [50]. Even a brief exposure to lists of words that share a sound pattern, such as words that are stressed on the first syllable, allows learners to use those shared patterns to find the words in a novel speech stream [41,46,47]. Given that phonotactic regularities can be used to segment the speech stream into word-like units, it may be necessary for learners to rapidly acquire phonotactic knowledge early in the learning process. If this is the case, then an expected learning trajectory would be one where phonotactic regularities are extracted from the speech stream first, before the individual words of the language are identified.

Finally, it is also possible that learners may rely on transitional probability cues as a direct route to both phonotactic regularities and word segmentation such that both regularities are acquired concurrently. Given that previous research has shown that learners can use transitional probability cues to track word boundaries [5,6] or the phonotactic patterns of words [13,15] in a continuous stream of speech, it is possible that learners use the transitional probability information as a route to both types of patterns. If transitional probability cues provide sufficient information to facilitate tracking both types of regularities, learners can extract both the individual word units and their phonotactic patterns simultaneously.

### The present study

The current study was designed to determine whether adult learners can segment a continuous stream of speech into words and learn the phonotactic pattern shared among a subset of these words based on a single stream of continuous speech. We additionally examined the manner in which the learning of both types of patterns unfolds by manipulating the duration of the exposure phase. Transitional probabilities between syllables in the speech stream were a strong cue to word boundaries in the language (1.0 within words and 0.15 between words). Furthermore, a nonadjacent phonotactic pattern structured a majority of the words. The speech stream contained 8 individual words, and 6 of the 8 words started with /t/ and ended with /u/. Thus, in contrast to previous studies [13], the phonotactic regularity was also a strong cue in the language, with 75% of the words in the language following the phonotactic pattern. Learners were tested not only on their acquisition of the specific individual words but also on their ability to generalize the phonotactic regularity to novel items not present in the speech stream.

We selected the phonotactic pattern to be similar to how phonotactic constraints are structured in natural languages (multiple sound features constrained to specific syllabic positions) but novel so as to minimize effects of language experience and mirror the situation of infants confronted with native language input (although we acknowledge that adults have a long history with their language(s) which may impact how they acquire regularities even from novel artificial materials, e.g., [51]). Further, we were careful to design the phonotactic pattern to be learnable given previous research. Adults can rapidly acquire the positional restrictions of phonemes (e.g., that they are word-initial or word-final) composing novel words [37,40]. Adults are also sensitive to nonadjacent phonological constraints and can acquire these constraints from brief exposures [23,33]. Furthermore, learners are particularly sensitive to phonotactic constraints at word edges [36].

To examine whether learners acquired both the phonotactics and the individual words of the language, we tested all participants on the learning of both regularities using foils that teased apart learning the individual words of the language from learning the phonotactic pattern. To assess how the learning of each pattern unfolded, we manipulated the amount of exposure to the language prior to testing across participants. Exposure length has been manipulated in only a limited set of studies examining statistical learning, providing a window into how learners acquire different types of regularities from the same set of input [23,27,34]. Given that learning one regularity may be dependent on learning the other, here we manipulate language exposure to assess the trajectory of learning each regularity. By decreasing the exposure length to the language, we attempt to capture the early phases of learning to examine if one regularity is learned first before the other.

Experiment 1 included three exposure durations (7 minutes, 3.5 minutes, and 2.5 minutes) and used a ratings test procedure to determine whether (1) learners could identify the words in the language and (2) learners could generalize the phonotactic pattern beyond the language materials. In Experiment 2, we replicated Experiment 1 by using a 2 alternative forced choice testing procedure (2AFC) after either 3.5 minutes of listening or 2.5 minutes of listening. If adults begin acquiring both types of regularities concurrently from the beginning of the acquisition process, then we should see evidence for learning both regularities even as exposure to the language is decreased. If, however, learning happens sequentially, then we would expect to see differences in learning outcomes for the two types of regularities as exposure to the language is decreased.

## Materials and methods

This study was approved by the University of Wisconsin-Madison IRB and the Arizona State University IRB (STUDY00007151). Written consent was obtained at UW-Madison and oral consent was obtained at Arizona State University.

### Experiment 1

The goal of Experiment 1 was to determine whether adults exposed to fluent speech could discover the individual words and the phonotactic pattern that characterized a subset of those words. The language consisted of eight bisyllabic words (Table 1). Six of the words (75%) were constructed with the same novel phonotactic pattern (initial /t/ and final /u/); the other two words did not follow the pattern. We manipulated the amount of exposure to the language at training between-participants, with participants listening to the language for 7 minutes, 3.5 minutes, or 2.5 minutes. During testing, adults were asked to rate the likelihood that individual items were words in the language. This method differs from most word segmentation studies, which typically employ 2-alternative forced choice (2AFC) tests: two items are presented back to back for each test trial, and learners are asked to choose which out of the two items is a word in the language. The 2AFC method provides a measure of the consistency with which individual participants choose one type of item as a word in the language over others. However, one constraint of this method is that it limits the number of types of items that can be compared to each other; most studies in the literature compare only two types of items, e.g., words vs. partwords. By using a rating scale instead, we were able to test each participant on a range of item types, allowing us to assess both type and token learning within participants.

**Table 1:** Stimuli for Experiments 1 and 2.

*Exposure items*	
Patterned Words	*tiepu*, *taylu*, *tafu*, *tehku*, *tohsu*, *teedu*
Unpatterned Words	*lehfay*, *keeda*
*Test Items*	
Patterned Words	*tohsu*, *teedu*
Unpatterned Words	*lehfay*,*keeda*
Partwords	*kuta*, *puteh*
Patterned Nonwords	*taydu*, *tiefu*
Additional Partwords (Exp. 2 only)	*dutay*, *lutoh*

The test items included both words from the exposure speech stream that followed the phonotactic pattern (Patterned Words) and words from the exposure speech stream that did not follow the phonotactic pattern (Unpatterned Words). Additionally, to test for knowledge of the novel phonotactic pattern, we included generalization items: novel combinations of syllables that conformed to the phonotactic pattern (Patterned Nonwords). Finally, we included Partword items: infrequent combinations of syllables spanning word boundaries that included the common sound features of the phonotactic pattern, but in the wrong positions (instead of initial /t/ and final /u/, the /u/ and /t/ occurred medially).

If learners successfully discovered the individual word units in the language, they should rate both Unpatterned Words and Patterned Words higher than Partwords. If learners successfully acquired the phonotactic pattern, they should rate the generalization items (Patterned Nonwords) higher than Partwords. We additionally compared item ratings to each other (Patterned Words to Unpatterned Words, and Patterned Words to Patterned Nonwords) to obtain further information about what participants learned. Finally, if participants prioritize these regularities differently at early versus later phases of learning, then the manipulation of exposure duration between subjects may indicate the order in which patterns are acquired.

#### Participants

Undergraduates (N = 133) were recruited from the subject pool at the University of Wisconsin-Madison. All participants were native English speakers; 46 participants indicated that they also spoke a second language. Participants were assigned to listen to the language for 7 minutes (N = 45), 3.5 minutes (N = 44) or 2.5 minutes (N = 44). Participants received course credit for participating in the study.

#### Stimuli

*Artificial language*. The artificial language consisted of 8 bisyllabic CVCV words (see Table 1). Six of the words–Patterned Words–followed a specific phonotactic pattern (they began with /t/ and ended in /u/). The other two words contained other word onsets and offsets (Unpatterned Words).

The stimuli were recorded by a female native English speaker in a monotone voice. To create the exposure speech stream, each syllable of each word was recorded in every possible context in which it would appear in the artificial language to preserve coarticulation and to ensure that acoustic cues to word boundaries were not provided, a method used previously by [52] (see also [53,54]). Each syllable was then manually extracted from each recording using Praat [55], normalized for pitch, duration, and intensity, and concatenated together into a continuous stream.

We manipulated the number of word repetitions in the exposure language to create different exposure lengths. The 7-minute language included 90 repetitions of each word, the 3.5-minute language included 45 repetitions of each word, and the 2.5-minute language included 30 repetitions of each word. The words were randomly ordered (with the constraint that the same word could not be presented twice in a row) and combined without any acoustic cues to the word boundaries. The only cue to the boundaries was the transitional probabilities (TPs) between syllables, which were 1.0 within words. TPs between words ranged between 0.08 and 0.22 with a mean of 0.15. The beginning and end of the recording was faded in and out to avoid any additional cues to word boundaries.

*Test items*. Four types of items were used for testing (see Table 1). Two Patterned Words and two Unpatterned Words were selected from the language to serve as the test words. Partwords were created by taking syllable combinations that spanned word boundaries. They thus included the individual segments from the phonotactic pattern presented during exposure, but in the incorrect positions (medial /ut/ sequence; e.g., *kuta*.) To test learning of the phonotactic pattern, we created novel words that followed the trained phonotactic pattern (Patterned Nonwords; initial /t/ and final /u/) by recombining the syllables in the Patterned Words not used during testing. The test items were recorded in isolation and were normed for pitch, intensity, and duration.

#### Procedure

Prior to the exposure phase, adults were asked to listen carefully to the language and told that they would be asked questions about it after. During the test phase, participants heard a sequence of test trials in which they were presented with individual test items and asked to rate, on a scale of 1 to 6, the likelihood that the item was a word in the language they just heard (1 = definitely not a word in the language; 6 = definitely a word in the language). The rating scale was presented on a number line for participants to click and confirm their rating. All test items were presented, in a random order, once in Block 1 and repeated a second time in Block 2. The experiment was designed and implemented using PsychoPy Builder [56]. Demographic and language questionnaires were administered after participants completed the experiment; the entire session lasted approximately 20 minutes.

### Experiment 2

We tested two additional groups of adults with a modified testing procedure: a 2AFC method designed to be more sensitive to individual participants’ learning. Word learning in Experiment 2 was assessed by comparing both types of Words versus Partwords. Additionally, by comparing performance across the two test types (Patterned Words/Partwords vs. Unpatterned Words/Partwords), we also were able to measure phonotactic learning. Although this design did not assess generalization, it did allow us to measure both learning of the individual words and the phonotactic pattern. If learners acquired the phonotactic pattern, then those words that include that pattern (Patterned Words) should be easier to discriminate from Partwords than those that do not contain the pattern (Unpatterned Words). Therefore, if adults learned the phonotactic regularity, we would expect performance on the Patterned Word tests to be higher than on the Unpatterned Word tests.

In Experiment 2, we sought to replicate Experiment 1 while gaining insight into what is being learned at the earliest phase of learning. To do so, we exposed adults to the language for either 3.5 minutes or 2.5 minutes. We predicted that, at least at 3.5 minutes of learning, adults should show evidence of having acquired both patterns from the language. Learning should therefore be above chance for both types of tests, with adults being more accurate on Patterned Word tests than Unpatterned Word tests. Performance after 2.5 minutes of exposure was assessed to allow us to determine what, if anything, adults acquire at the earliest phase of learning.

#### Participants

A new group of native English-speaking undergraduates participated in Experiment 2 for course credit (N = 93), with 39 participants indicating they spoke a second language. One group of participants were assigned to listen to the language for 3.5 minutes (N = 48, 23 recruited from the University of Wisconsin subject pool, and 25 recruited from the Arizona State University subject pool) while a second group was assigned to listen to the language for 2.5 minutes (N = 45, all recruited from the University of Wisconsin subject pool).

#### Stimuli, design, and procedure

Participants in Experiment 2 were presented with either 3.5 or 2.5 minutes of exposure to the language. We made two design changes to testing. First, we changed the testing procedure to forced choice. Second, we only included Unpatterned Words, Patterned Words, and Partwords in the test phase. In order to control for the number of repetitions of each test item, we increased the number of Partword tokens relative to Experiment 1 (see Table 1).

On each test trial, participants were presented with two items, one right after the other, and asked to choose which of the two items was a word in the exposure language. Each trial contrasted a single Word with a single Partword. The materials included two Unpatterned Words, two Patterned Words, and four Partwords (see Table 1). All Words were paired with all Partwords, for a total of 16 test trials. Each item occurred equally often during testing. Order of presentation of the paired items was counterbalanced across trials. The test trials were blocked such that each pairing was presented once (randomly ordered) within a block.

## Results

### Experiment 1

We analyzed participants’ trial-by-trial ratings to the different items by estimating linear mixed effects models (fit using Maximum Likelihood Estimation) and comparing these models using Wald Chi-square tests of best fit. All analyses were conducted in R (version 3.4.3) using the package lme4 (version 1.1–21; [57]) and we report Chi-square statistics for all analyses. All data and the R-script for the main analyses reported in this manuscript are available at: https://osf.io/4tc8v/.

**Fig 1:**
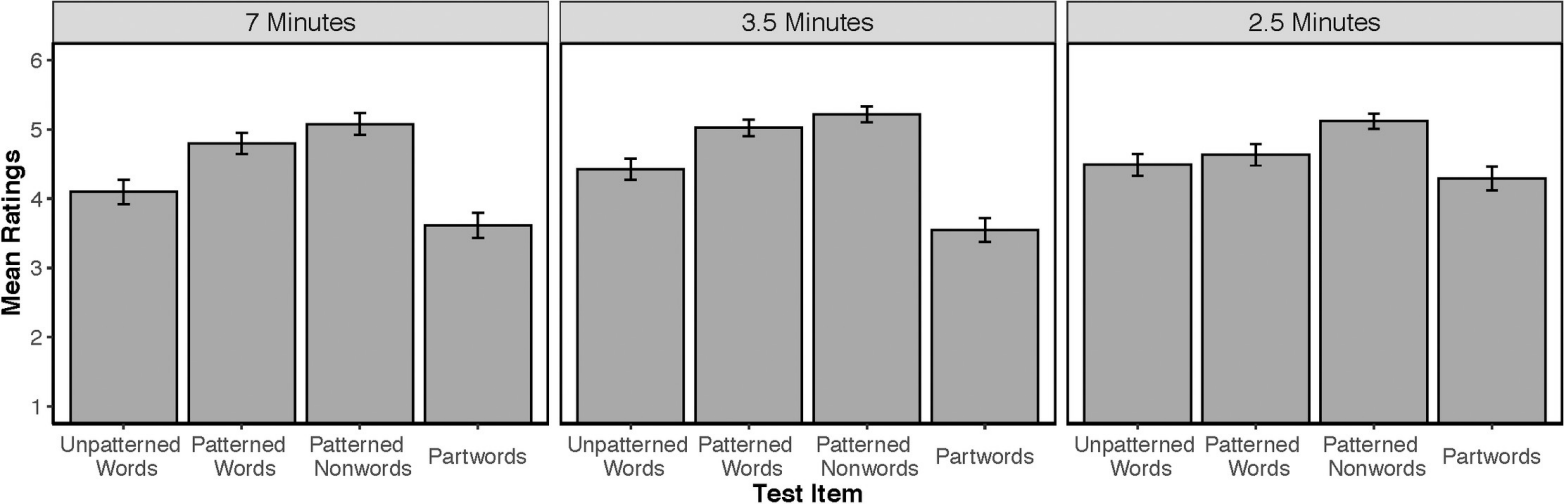
Ratings by exposure length in Experiment 1. Mean ratings (and standard error of the mean) for test items presented in Experiment 1 for language exposure times of 7 minutes, 3.5 minutes, and 2.5 minutes.

Fig 1 displays the mean ratings for all test items for each exposure length separately. We first conducted a model that included the fixed effects of word type (with Partwords set as the reference category), exposure length (with 7 minutes set as the reference category), test block, and second language exposure, as well as a by-subject random slope for the interaction between word type and block, and a by-item random slope for the interaction between word type, block, and exposure. After this model resulted in a singular fit, we reduced the random effect structure until identifying a model that did not result in a singular fit or in problems of convergence (according to [58]). We did this by first removing exposure, block, and word type (in that order) in the by-item random slope, and then block and word type (in that order) in the by-subject random slope. The final model without singularity or convergence issues included only random intercepts for subject and item. Although results were qualitatively the same across all models, the effects of exposure did vary somewhat. All model results can be found in the Appendix (S1 Appendix). Results of our final model showed a significant effect of word type (χ^2^(3) = 16.19, p = 0.001), a significant effect of exposure length (χ^2^(2) = 9.16, p = 0.01), and a significant interaction between exposure length and word type (χ^2^(6) = 23.9, p<0.001). No other effects or interactions were significant.

To better understand our significant effects, we used dummy coding to conduct pairwise comparisons in our final model. First, we examined the significant effect of word type by comparing all items to Partwords. Patterned Words (M = 4.82, SD = 0.6) and Patterned Nonwords (M = 5.14, SD = 0.84) were each rated significantly higher than Partwords (M = 3.82, SD = 1.21), demonstrating overall successful learning of the patterned words in the speech stream as well as generalization of the phonotactic pattern (Patterned Words vs. Partwords: b = 1.2, STE = 0.29, χ^2^(1) = 33.76, p<0.001; Patterned Nonwords vs. Partwords: b = 1.54, STE = 0.39, χ^2^(1) = 65.67, p<0.001). Ratings for Unpatterned Words (M = 4.34, SD = 1.1) were marginally higher than ratings for Partwords (b = 0.70, STE = 0.39, χ^2^(1) = 3.2, p = 0.07). Finally, to further assess what participants may have learned, we compared each item to Patterned Words. There was no significant difference between Patterned Words vs. Unpatterned Words (b = -0.50, STE = 0.39, χ^2^(1) = 1.61, p = 0.20) and Patterned Words vs. Patterned Nonwords (b = 0.37, STE = 0.39, χ^2^(1) = 0.74, p = 0.39).

We next examined the significant effect of exposure length as well as the interaction between exposure length and word type using dummy coding to conduct pairwise comparisons. When comparing 7 minutes exposure to 3.5 minutes exposure groups, there were no significant differences in overall ratings between the two [b = -0.16, STE = 0.29, χ^2^(1) = 0.29, p = 0.59] and only a marginally significant exposure x item type interaction [χ^2^(3) = 7.74, p = 0.052]. When comparing performance for the 7 minutes group to the 2.5 minutes group, there was a significant effect of exposure length on overall ratings performance [b = 0.67, STE = 0.29, χ^2^(1) = 5.35, p = 0.02], and a significant interaction between exposure and word type [exposure x word type: [χ^2^(3) = 8.8, p = 0.03]. When comparing the 3.5 minutes to 2.5 minutes exposure times, there was a significant overall effect of exposure length on ratings [b = -0.83, STE = 0.29, χ^2^(1) = 8.1, p = 0.004] and a significant exposure x item type interaction [χ^2^(3) = 19.3, p<0.001].

These results indicate that performance for the 2.5 minutes exposure group was most different than all other exposure groups. Specifically, the 2.5 exposure group had overall lower ratings and additionally, rated only the Patterned Nonwords (novel sequences following the familiar phonotactic pattern) higher than Partwords. We report the pair-wise comparisons for each word type for each exposure length separately demonstrating these differences.

*7 minutes of exposure*. Adults successfully learned the phonotactic pattern, rating Patterned Words (M = 4.8, SD = 1.2) and Patterned Nonwords (M = 5.07, SD = 1.03) higher than Partwords (M = 3.61, SD = 1.23; Patterned Words vs. Partwords: b = 1.34, STE = 0.49, χ^2^(1) = 7.31, p = 0.007; Patterned Nonwords vs. Partwords: b = 1.52, STE = 0.49, χ^2^(1) = 9.44, p = 0.002). However, ratings for Unpatterned Words (M = 4.1, SD = 1.20) were not significantly different than ratings for Partwords (b = 0.40, STE = 0.49, χ^2^(1) = 0.65, p = 0.42). Patterned Words were rated marginally higher than Unpatterned Words (b = -0.94, STE = 0.49, χ^2^(1) = 3.61, p = 0.057) but were not rated differently from Patterned Nonwords (b = 0.18, STE = 0.49, χ^2^(1) = 0.13, p = 0.71).

#### 3.5 minutes of exposure

Adults successfully learned both types of words in the language [Unpatterned Words (M = 4.43, SD = 1.02) vs. Partwords (M = 3.55, SD = 1.15): b = 1.41, STE = 0.40, χ^2^(1) = 12.66, p<0.001; Patterned Words (M = 5.02, SD = 0.79) vs. Partwords: b = 1.90, STE = 0.40, χ^2^(1) = 22.78, p<0.001]. Additionally, adults successfully learned the phonotactic pattern, as shown by a higher mean rating for Patterned Nonwords (M = 5.22, SD = 0.75) compared to Partwords [b = 2.10, STE = 0.40, χ^2^(1) = 28.02, p<0.001]. Finally, Patterned Words were rated similarly to Unpatterned words [b = -0.48, STE = 0.40, χ^2^(1) = 1.48, p = 0.22] and Patterned Nonwords [b = 0.21, STE = 0.40, χ^2^(1) = 0.27, p = 0.60].

#### 2.5 minutes of exposure

Unlike the longer exposure lengths, only Patterned Nonwords (M = 5.12, SD = 0.72) were rated significantly different than Partwords (M = 4.3, SD = 1.14) [b = 0.98, STE = 0.47, χ^2^(1) = 4.46, p = 0.03]. All other items were rated similarly to Partwords [Unpatterned Words: M = 4.49, SD = 1.04; b = 0.29, STE = 0.47, χ^2^(1) = 0.40, p = 0.53; Patterned Words: M = 4.63, SD = 1.02; b = 0.36, STE = 0.47, χ^2^(1) = 0.61, p = 0.53]. When all items were compared to Patterned Words, the results showed that Patterned Nonwords were rated higher than Patterned Words [b = 0.62, STE = 0.47, χ^2^(1) = 6.0, p = 0.01]; Unpatterned Words were rated similarly to Patterned Words [b = -0.07, STE = 0.47, χ^2^(1) = 0.06, p = 0.81].

To summarize the results of Experiment 1, as a group, adults succeeded at rating words in the language composed of the phonotactic pattern higher than partwords. As a group, adults also rated novel items containing the pattern higher than partwords. These results suggest that adults learned the phonotactic pattern and generalized that pattern to novel items. Unpatterned words were only marginally rated higher than partwords for all participants combined. However, the 3.5 minute exposure group rated all types of words (Unpatterned Words, Patterned Words, and Patterned Nonwords) higher than partwords, suggesting that learning for each of these patterns does not compete: adults are capable of acquiring types and tokens from the same speech stream. The fact that Unpatterned words were only marginally rated higher than Partwords for our exposure groups combined is likely due to differences between exposure lengths, which we discuss next.

A second question focuses on the relationship between the acquisition of the two types of patterns. Is one type of information acquired before the other, or are they both acquired simultaneously? To address this question, we examined the learning of each type of pattern at the different exposure lengths, focusing mainly on the 2.5 minute exposure group, as this group was significantly different from the other exposure groups. Learning of the words without the phonotactic pattern did not occur at the 2.5 minute exposure length, but did occur at the 3.5 minutes exposure length and did not occur at the 7 minute exposure length (though we note there was no difference between the 3.5 and 7 minute exposure groups), suggesting the learning of the Unpatterned words was the most fragile. Learning of the words with the phonotactic pattern was not present at the 2.5 minute exposure length but was present at the two longest exposure lengths, at 3.5 minutes and 7 minutes. Finally, successful generalization of the phonotactic pattern was present at all three exposure lengths, suggesting that the abstraction of the phonotactic pattern was most robust. These results point to a few different possibilities with respect to how learners navigate the acquisition of the two different patterns. The first possibility is that phonotactic learning may come first—with 2.5 minutes of exposure, adults rated Patterned Nonwords higher than Partwords. This suggests that adults learned the phonotactic pattern successfully without segmenting the individual words of the language. One caveat, however, is that although adults showed evidence of generalization, they did not rate Patterned Words, items in the language composed of the phonotactic pattern, higher than Partwords. How can adults show successful generalization without successful identification of the tokens in the language that contain the phonotactic pattern? On the one hand, adults may have represented the phonotactic pattern more strongly than the individual items in the language even if they contained the phonotactic pattern. This possibility is supported by a trend for ratings for Patterned Nonwords to be higher than Patterned Words present in all three exposure groups (though we note that this difference was only significant at the 2.5 minute exposure length). This possibility is also supported by the fact that Unpatterned items were rated higher than Partwords for only one of the three exposure groups, and as a group, our participants rated Unpatterned words only marginally higher than partwords. On the other hand, adults may not have fully learned the phonotactic pattern, as the representation of the pattern should have yielded higher ratings for all of the items containing the pattern. Thus, at the 2.5 minute exposure length, adults seemed to generalize the phonotactic pattern found in the language, but it is not clear if their representation of that phonotactic pattern includes knowledge of the individual words of the language.

One possible reason for the inconclusive results at 2.5 minutes of exposure is that at such brief exposure to a language containing 8 words, adults may either not be capable of learning either regularity, or may only be weakly representing both regularities. If it is the case that at a shorter exposure, learners are representing patterns weakly, the ratings procedure may not be sensitive enough to capture this weak learning. Further, only the 3.5 minute exposure group rated Unpatterned words significantly higher than partwords. Therefore, it is also important to further examine if learners are indeed able to track the individual words in the language that do not follow the phonotactic pattern.

In Experiment 2, we used a different testing method, 2AFC, to 1) replicate the result that adults can learn both words and phonotactics by 3.5 minutes of listening time, and 2) better understand whether adults are learning the words and the phonotactic pattern at the shortest exposure time of 2.5 minutes. Using a 2AFC test measure allows participants to choose between two types of items for multiple test trials. The 2AFC method therefore provides a measure of how consistently individual participants choose one type of item as a word in the language over others. 2AFC testing is frequently used in assessments of word segmentation and can capture meaningful individual differences in participants’ ability to track patterns in the speech stream. For example, 2AFC assessments of learners’ performance on word segmentation tasks tend to be stable within individuals [59], and are related to individual language abilities [60]. In Experiment 2, we presented adults with the language for either 3.5 minutes of exposure or 2.5 minutes of exposure, but this time tested them using the 2AFC procedure. In order to use the 2AFC method, which limits the number of possible comparisons due to test duration, we constrained our items to contrast the two types of Words (Patterned and Unpatterned) against Partwords. Importantly, although this design does not test generalization, it does allow us to examine if adults are learning any of the words of the language and the phonotactic pattern.

### Experiment 2

**Fig 2:**
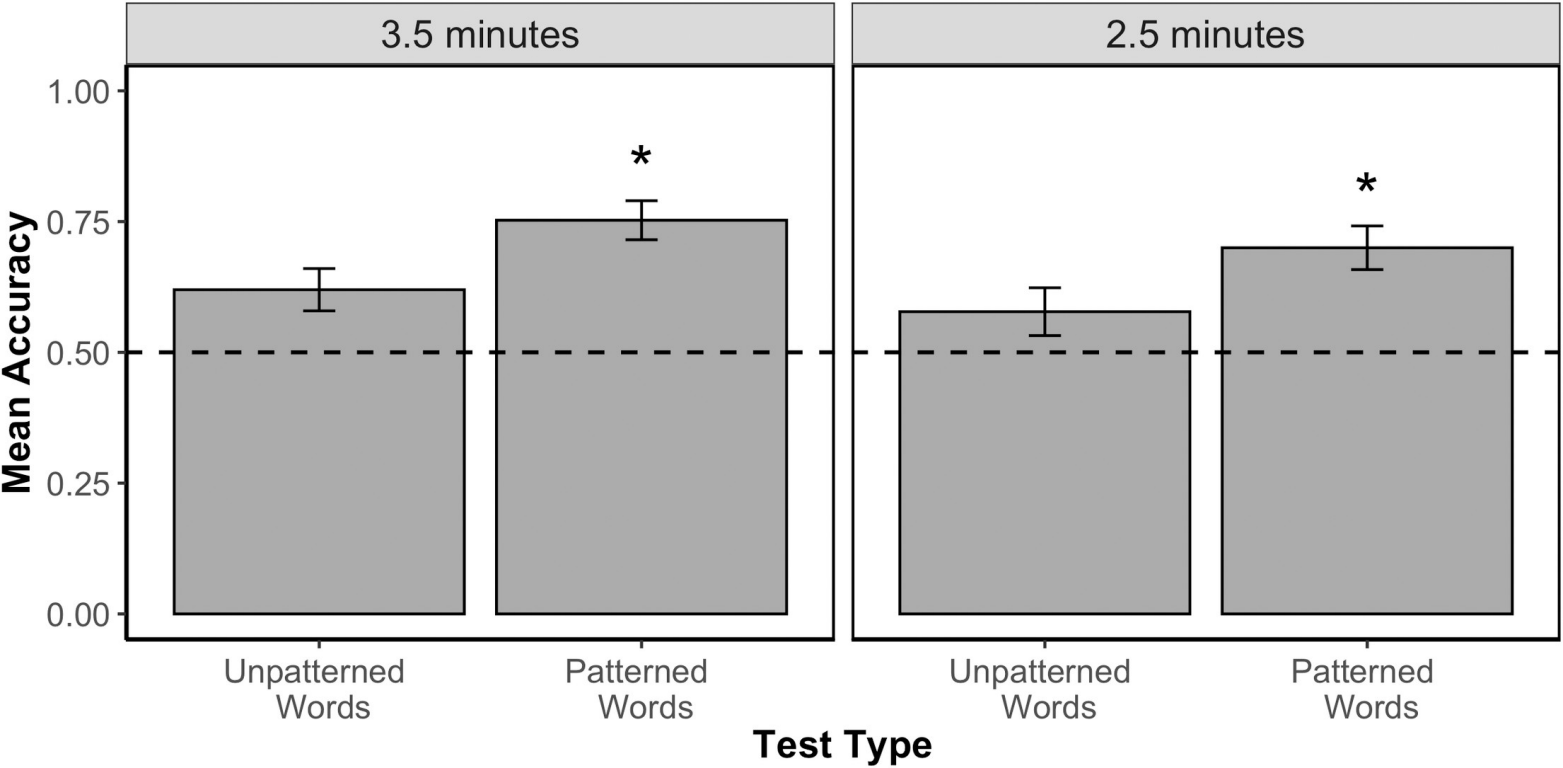
Accuracy by exposure length in Experiment 2. Mean proportion correct (and standard error of the mean) for Word vs. Partword test items presented in Experiment 2 after 3.5 minutes or 2.5 minutes of exposure to the language. Dotted line indicates chance performance (0.5). Asterisk denotes significant difference from chance performance.

Fig 2 displays participants’ mean accuracy scores for each test type for each exposure length. We first analyzed participants’ trial-by-trial test accuracy scores (1 or 0) for each test type using a logistic mixed effects model that included fixed effects for test type, exposure length, block, and second language exposure, a by-subject random slope for the interaction between test type and block, and a by-item random slope for the interaction between test type, block, and exposure. When this model failed to converge, we reduced the random effect structure by first removing the interaction terms in the by-item random slope (exposure, then block, then test type) and then removing the interaction terms in the by-subject random slope (block then test type). A model with random intercepts for subject and item still failed to converge. We then opted for reducing the complexity of the original model by taking out the fixed effects of block and second language exposure after we verified that, as in Experiment 1, these factors were not linked with accuracy scores in Experiment 2 (all model results can be found in the S1 Appendix). Our final model included the fixed effects of test type and exposure length, and random intercepts for subject and item.

Results of the final model revealed only a significant effect of test type [χ^2^(1) = 9.06, p = 0.003]. There was no significant effect of exposure length [χ^2^(1) = 0.37, p = 0.54] and no significant interaction between exposure length and test type [χ^2^(1) = 0.19, p = 0.66]. Overall, participants were more accurate on Patterned Word tests (M = 0.73, SD = 0.27) than Unpatterned Word tests (M = 0.60, SD = 0.29). Overall, participants were successful at learning for both Unpatterned Word tests (comparison to chance: χ^2^(1) = 6.20, p = 0.01) and for Patterned Word tests (comparison to chance: χ^2^(1) = 34.6, p<0.001).

Although there were no effects of language exposure length, we report the results for each group separately below for consistency with Experiment 1.

#### 3.5 minutes of exposure

Results revealed a significant advantage for Patterned Word tests (M = 0.75, SD = 0.26) over Unpatterned Word tests [M = 0.62, SD = 0.28; χ^2^(1) = 4.64, p = 0.03]. Participants’ accuracy scores were above chance for Patterned Word tests [χ^2^(1) = 19.12, p<0.001] but only marginally above chance for Unpatterned Word tests [χ^2^ (1) = 3.70, p = 0.05].

#### 2.5 minutes of exposure

Again, there was a significant advantage for Patterned Word test items (M = 0.70, SD = 0.28) compared to Unpatterned Word test items [M = 0.58, SD = 0.31; χ^2^(1) = 15.30, p<0.001; we note that for this analysis, the model only included a random intercept for subject after a model including a random intercept for subject and item failed to converge]. Further, similarly to the data from the 3.5 minutes of exposure group, participants performed above chance only on the Patterned Word test items [χ^2^(1) = 21.9, p<0.001]. Adults were marginally above chance on the Unpatterned Word test items [χ^2^(1) = 3.47, p = 0.06; here again we only included a random intercept for subject after a model including a random intercept for subject and item failed to converge].

As a group, adults demonstrated evidence of tracking the words and their phonotactic features from a continuous speech stream. Specifically, adults discriminated both Unpatterned Words and Patterned Words from Partwords, showing that adults tracked the individual words in the language. Additionally, there was a performance advantage for Patterned Words tests over Unpatterned Word tests, revealing that participants also tracked the phonotactic pattern. Unlike Experiment 1, there was no significant effect of exposure group, suggesting that there were no strong differences in learning when listening to the language for 2.5 minutes compared to 3.5 minutes.

When examining each exposure group separately, results showed learning of the Unpatterned words was only marginally above chance performance. This result suggests that the phonotactic regularity was most strongly represented in learning across our exposure groups. However, we did find that our participants in Experiment 2 as a group showed learning of all words in the language in addition to the phonotactic pattern. This pattern of results reveals that adults are capable of learning both individual words and phonotactics from the same speech stream.

### Exploratory analyses

Although we found evidence for learning both words and phonotactics in Experiment 1 and Experiment 2, several null effects make it difficult to draw strong conclusions about the trajectory of learning these two patterns: specifically, the null results of exposure in Experiment 1 (comparison of 3.5 minutes and 7 minutes) and in Experiment 2 (comparison of 2.5 minutes and 3.5 minutes). We examined these null results further by calculating Bayes factors to assess if there was significant evidence against an effect of exposure in both comparisons (see S2 Appendix). We note that these analyses are exploratory and should be interpreted with caution.

We first explored the null effects of exposure in Experiment 1 (3.5 minutes vs. 7 minutes) by conducting a repeated measures Bayesian ANOVA. Results indicated moderate evidence against an effect of exposure and strong evidence against an interaction between exposure and word type (S2 Appendix). When assessing the null effects of exposure (using again a repeated measures Bayesian ANOVA) in Experiment 2 (2.5 minutes vs. 3.5 minutes), results demonstrated weak evidence against an effect of exposure, and moderate to strong evidence against an interaction between exposure and word type (S2 Appendix). These findings suggest there was no difference in performance when listening to the language for 3.5 minutes compared to 7 minutes in Experiment 1, and 2.5 minutes vs. 3.5 minutes in Experiment 2.

## Discussion

Our first research question was whether learners were capable of tracking regularities at two different levels of abstraction, individual words and a phonotactic pattern shared by a subset of those words, from a single input stream. Using both ratings (Experiment 1) and 2AFC (Experiment 2) testing methods, adults showed evidence of successfully extracting the phonotactic pattern from the same speech stream. We also found evidence that adults were capable of tracking the individual words of the language (with or without the pattern) when they listened to the language for 3.5 minutes in Experiment 1, and for the combined groups (2.5 minutes and 3.5 minutes) in Experiment 2. These results suggest that adults can rapidly extract both types of patterns from the same input.

Our second research question focused on whether one regularity was tracked before the other. In Experiment 1, using a ratings test procedure, we found that although at 3.5 minutes of listening, adults showed evidence of tracking both types of regularities, at 2.5 minutes of listening, they only showed evidence of generalizing the phonotactic pattern. With 2.5 minutes of listening time, there was no evidence that adults learned any of the individual words of the language. In contrast, in Experiment 2, we found that after 2.5 minutes of listening time, adults were successful at learning the words in the language containing the phonotactic pattern, but did not succeed at learning the words without the phonotactic pattern.

These findings point to the possibility that the phonotactic regularity was learned first, without segmenting the language. However, inconsistencies across experiments make it difficult to make strong conclusions about what type of learning is occurring at the shortest exposure length. First, the difference between the two shortest exposure lengths (2.5 minutes vs. 3.5 minutes) was present in Experiment 1 and not in Experiment 2. Further, at the shortest exposure length, learning of patterned words was not present in Experiment 1 but was present in Experiment 2. What is clear is that by the time learners have received 3.5 minutes of exposure, they have tracked both individual words and phonotactic regularities from the same continuous stream of speech–this result was observed in both Experiment 1 (for the 3.5 minute exposure group) and Experiment 2 (for the 2.5 and 3.5 exposure groups combined). Thus, a second and more likely possibility with respect to how learners navigate acquiring both patterns is that learners acquire words and phonotactics simultaneously, but initially weakly, only showing clear evidence of learning after enough exposure (at least 3.5 minutes) to the language. Learners may therefore use transitional probability cues as a direct route to tracking the phonotactic pattern and the individual words in the speech stream. Future studies with very brief listening times will be helpful to better understand what adults may be tracking during the initial phases of learning, especially given that recent research suggests that adult learners are capable of extracting word regularities from continuous speech from as little as 2 repetitions [61].

Our study is the first to examine acquisition of both words and phonotactics in the same learner, demonstrating that adults can acquire both types of patterns from the same speech stream by 3.5 minutes of listening time. Although previous research shows that learners can abstract rules about specific speech input and generalize those rules to novel situations [13–15,23–30], only a few studies have examined the ability to both extract words and learn rules from the same continuous stream of speech, demonstrating that learners have biases with respect to tracking patterns at different levels of abstraction [14,27,30]. Our findings suggest a different picture: when faced with novel regularities at two levels of abstraction (token and type level information), learners are able to represent both types of regularities rapidly. Our study does differ from previous studies in a number of ways. It is therefore possible that learners’ ability to track regularities at different levels of abstraction may depend on the specifics of the learning problem. For example, the probability of both the token-level and type-level patterns was strong. Transitional probability cues were 1.0 for Words and an average of 0.15 for Partwords, making transitional probability cues a strong signal to the individual words in the speech stream. The phonotactic pattern presented in the speech stream was also strong, occurring with a high probability (it was present in 75% of the words in the language). Cue strength modulates how learners track the statistical properties of speech [62–64]. A strong signal to word boundaries, and a highly probable phonotactic pattern, may indeed have allowed learners in our study to rapidly acquire both regularities. An avenue for future research is to examine if manipulating the strength of the patterns at different levels of abstraction shifts whether adults are capable of tracking type- and token-level information simultaneously.

An additional difference from previous research is that our language contained adjacent transitional probabilities to signal the words in the language at the same time as a nonadjacent phonotactic pattern. Thus, in addition to having to track regularities at multiple levels of abstraction, learners also had to track regularities with differing distributional structure. The fact that adults were successful at tracking both regularities from the same speech stream is consistent with recent findings showing that adults are capable of tracking both adjacent and nonadjacent regularities simultaneously [34,35]. Importantly, our work extends this previous research by demonstrating that adults can track both adjacent and nonadjacent regularities from speech that is unsegmented. It is also important to note that the type of distributional structure of the regularities signaling word boundaries and the regularities signaling phonotactic patterns may modulate if both regularities can be learned. Previous research has demonstrated that infants and adult learners are successful at tracking nonadjacent dependencies, but only under certain conditions [31–33]. For example, when presented with a continuous stream of speech, adults failed at tracking nonadjacent dependencies between syllables, but succeeded at tracking nonadjacent dependencies between consonants or vowels [33]. The difficulty of tracking nonadjacent syllable statistics may have led to a failure to track both words and the rules composing those words from unsegmented speech in previous research [27]. Given that phonotactic patterns can be composed of adjacent and nonadjacent regularities, future work should manipulate the distributional structure of the regularities signaling words and phonotactics to better understand how these factors affect learning both from the same speech stream.

In terms of what is learned at the earliest time point tested (2.5 minutes of listening time), our results were mixed. One potential reason for the inconsistent group data found at the earliest phase of learning tested is that there may be individual differences with respect to how learners navigate acquiring both types of regularities. In support of the notion that learners may differ in how they extract statistical regularities from continuous input, Siegelman, Bogaerts, Armstrong, & Frost [65] assessed whether adult participants extracted local clusters (co-occurrences between individual elements) or global clusters (chunk-like information about which sets of elements go together) from a stream of visual shapes containing transitional probability information to signal which shapes follow each other. They found that both strategies were present in learning, with some learners extracting local clusters and others global clusters. Our study was not set up to assess if learners differ in what pattern they are extracting from the input, but an exploratory examination of the 2.5 minute exposure group in Experiment 2 suggests there may be individual differences: 20 participants were above 0.5 accuracy for both the Unpatterned words and Patterned words, 11 participants were above 0.5 accuracy for only the Patterned words, 5 participants were above 0.5 accuracy for only the Unpatterned words, and 9 participants were at or below 0.5 accuracy for both tests. Many adults seemed to have learned both types of words rapidly, while others seem to have only learned one type of word (mainly the words containing the phonotactic pattern). Thus, different participants may have implemented different strategies for acquiring the patterns at the shortest exposure time to the language. By implementing a testing procedure that more consistently measures knowledge acquired by each participant [66], future research can better assess if there are individual differences with respect to what regularities participants extract from the same input, particularly at the earliest phases of learning.

Although our study only tested adults, it is important to consider the developmental trajectory of discovering words and phonotactic patterns from continuous speech. Infants rapidly abstract adjacent and nonadjacent phonotactic patterns in their native language by 10 months of age, before they have fully acquired the vocabulary of their language [8–12]. However, infants demonstrate nascent understanding of the words in their native language at a very young age [16,17]. Infants have likely segmented many candidate words during their first 9 months, even if they have not yet mapped them to meanings [18]. Further, knowledge of phonotactic regularities in the native language is related to vocabulary knowledge in young infants [67]. Thus, it is possible that infants may track both the individual words of their native language while simultaneously abstracting their languages’ phonotactic structure. Although our results provide some support for the ability of learners to track both patterns together, it is difficult to extend our results to infant learning, given that pattern abstraction and generalization may occur differently across the two age groups [68,69]. Examining these issues directly by testing infants and children should further provide insights into how the learning of these trajectories unfolds over development.

In conclusion, we examined whether and how adult learners track two types of regularities (the individual words and their phonotactics) from a continuous stream of speech containing only transitional probabilities as a cue to word boundaries. Our findings suggest that adults can track both regularities even from relatively brief exposures to the speech stream. Together, our results suggest that adults can concurrently extract regularities at multiple levels of abstraction from a single set of language input.

## Supporting information

S1 Appendix Results of models with singularity or convergence issues in Experiment 1 and Experiment 2. (PDF)

S2 Appendix Exploratory Bayesian analyses to assess null effects of language exposure length. (PDF)
