# Supplementary material for: Two for the price of one: Concurrent learning of words and phonotactic regularities from continuous speech
Source: PLoS One. 2021 Jun 11;16(6):e0253039. doi: 10.1371/journal.pone.0253039 (PMC8195377; doi:10.1371/journal.pone.0253039)
Supplement: S1 Appendix — (PDF) [file pone.0253039.s001.pdf]

S1 Appendix

Experiment 1 results of models with singularity or convergence issues

All linear mixed effects models in included the fixed effects of word type, exposure, block, and second language. Each model included a different random effect structure – we began with the maximal structure and reduced it by dropping terms as indicated for each model.

Full model. This model included a by-subject random slope for the interaction between word type and block, and a by-item random slope for the interaction between word type, exposure, and block. This model resulted in a singular fit. Results showed a significant effect of word type ($\chi^2(3)=9.4$, $p=0.02$), and a marginal effect of exposure ($\chi^2(2)=5.87$, $p=0.05$). No other effects or interactions were significant.

Reduced model 1. This model included a by-subject random slope for the interaction between word type and block, and a by-item random slope for the interaction between word type and exposure (block was dropped). This model resulted in a singular fit. Results showed a significant effect of word type ($\chi^2(3)=11.10$, $p=0.01$), and a marginal effect of exposure ($\chi^2(2)=5.02$, $p=0.08$). No other effects or interactions were significant.

Reduced model 2. This model included a by-subject random slope for the interaction between word type and block, and a by-item random slope for word type (exposure was dropped). This model resulted in a singular fit. Results showed a significant effect of word type ($\chi^2(3)=13.48$, $p=0.004$), a significant effect of exposure ($\chi^2(2)=6.24$, $p=0.04$), and a marginal interaction between exposure and word type ($\chi^2(6)=12.5$, $p=0.05$). No other effects or interactions were significant.

Reduced model 3. This model included a by-subject random slope for the interaction between word type and block, and a random intercept for item (word type was dropped). This

model resulted in a singular fit. Results showed a significant effect of word type ($\chi^2(3)=13.0$, $p=0.005$), a significant effect of exposure ($\chi^2(2)=6.21$, $p=0.04$), and a significant interaction between exposure and word type ($\chi^2(6)=12.60$, $p=0.0498$). No other effects or interactions were significant.

Reduced model 4. This model included a by-subject random slope for word type (block was dropped) and block, and a random intercept for item. This model failed to converge. Results showed a significant effect of word type ($\chi^2(3)=13.4$, $p=0.004$), a significant effect of exposure ($\chi^2(2)=6.26$, $p=0.04$), and a significant interaction between exposure and word type ($\chi^2(6)=13.86$, $p=0.03$). No other effects or interactions were significant.

Experiment 2 results of models with singularity or convergence issues

All logistic mixed effects models in included the fixed effects of test type, exposure, block, and second language except where otherwise noted. Each model included a different random effect structure – we began with the maximal structure and reduced it by dropping terms as indicated for each model.

Full model. This model included a by-subject random slope for the interaction between test type and block, and a by-item random slope for the interaction between test type, exposure, and block. This model failed to converge. Results showed no significant effects (test type: $\chi^2(1)=1.27$, $p=0.26$; exposure: χ^2 $(1)=0.96$, $p=0.33$; test type x exposure: χ^2 $(1)=0.002$, $p=0.96$).

Reduced model 1. This model included a by-subject random slope for the interaction between test type and block, and a by-item random slope for the interaction between test type and exposure (block was dropped). This model failed to converge. Results showed no significant effects (test type: $\chi^2(1)=1.19$, $p=0.27$; exposure: χ^2 $(1)=1.3$, $p=0.25$; test type x exposure: χ^2 $(1)=0.04$, $p=0.84$).

Reduced model 2. This model included a by-subject random slope for the interaction between test type and block, and a by-item random slope for test type and exposure (exposure was dropped). This model failed to converge. Results showed no significant effects (test type: $\chi^2(1)=2.05$, $p=0.15$; exposure: χ^2 $(1)=1.25$, $p=0.26$; test type x exposure: χ^2 $(1)=0.02$, $p=0.88$).

Reduced model 3. This model included a by-subject random slope for the interaction between test type and block, and a random intercept for item (test type was dropped). This model failed to converge. Results showed no significant effects (test type: $\chi^2(1)=1.96$, $p=0.16$; exposure: χ^2 $(1)=1.23$, $p=0.27$; test type x exposure: χ^2 $(1)=0.03$, $p=0.87$).

Reduced model 4. This model included a by-subject random slope test type (block was dropped), and a random intercept for item. This model failed to converge. Results showed no significant effects (test type: $\chi^2(1)=1.7$, $p=0.19$; exposure: χ^2 $(1)=1.4$, $p=0.24$; test type x exposure: χ^2 $(1)=0.04$, $p=0.84$).

Reduced model 5. This model included a random intercept for subject and random intercept for item. This model failed to converge. Results showed no significant effects (test type: $\chi^2(1)=1.74$, $p=0.19$; exposure: χ^2 $(1)=1.23$, $p=0.27$; test type x exposure: χ^2 $(1)=0.02$, $p=0.90$).

Assessing effects of block and second language exposure. Given that all models failed to converge, we decided to simplify the model further by dropping two fixed effects less relevant to our hypotheses: block and second language exposure. Before doing so, we verified that these two factors were not linked to performance. To assess this, we conducted a logistic mixed effects model with block and second language exposure as fixed effects, and random intercepts for subject and item. Results showed no significant effects (block: $\chi^2(1)=1.23$, $p=0.28$; second

language exposure: $\chi^2(1)=0.0004$, $p=0.98$; block x second language exposure: $\chi^2(1)=0.06$, $p=0.81$).
