# Supplementary material for: Two for the price of one: Concurrent learning of words and phonotactic regularities from continuous speech
Source: PLoS One. 2021 Jun 11;16(6):e0253039. doi: 10.1371/journal.pone.0253039 (PMC8195377; doi:10.1371/journal.pone.0253039)
Supplement: S2 Appendix — (PDF) [file pone.0253039.s002.pdf]

S2 Appendix

Bayesian analyses were conducted using JASP. All results output can be found at OSF [https://osf.io/4tc8v/].

Experiment 1, 3.5 minutes vs. 7 minutes.

We compared the 3.5 minute exposure group to the 7 minute exposure group in Experiment 1 by conducting a repeated measures Bayesian ANOVA on the data aggregated over trial and item. We note this differs from our approach to use linear mixed methods analyses on the trial by trial data, and so before calculating bayes factors, we first verified that a traditional repeated measures ANOVA on the aggregated data yielded results consistent to our linear mixed methods results. The traditional repeated measures ANOVA revealed a significant main effect of word type ($F(3,87)=50$, $p>0.001$). There was no significant effect of exposure ($F(1,87)=1.33$, $p=0.25$) and no significant interaction between exposure and word type ($F(3,87)=0.7$, $p=0.55$).

The repeated measures Bayesian ANOVA showed that a model that included only the effect of word type was the best performing model. When comparing all other models to the word type only model, results showed moderate evidence against a model that added exposure (BF10=0.284), strong evidence against a model that added exposure and the interaction between word type and exposure (BF10=0.020) and extremely strong evidence against a model that included only exposure (BF10<0.01). Comparison of the different effects revealed overwhelming evidence for the inclusion of word type (BFIncl = 1e+23), moderate evidence against inclusion of exposure (BFIncl = 0.284) and strong evidence against inclusion of the interaction between word type and exposure (BFIncl = 0.070). Both methods of model comparison demonstrate evidence against an interaction between exposure and word type.

Experiment 2, 2.5 minutes vs. 3.5 minutes.

In Experiment 2, comparing the 3.5 minute exposure and 2.5 minute exposure groups using a traditional repeated measures ANOVA aggregated over trial and item revealed a significant effect of word type ($F(1,91)=21.34$, $p<0.001$). There was no significant effect of exposure ($F(1,91)=0.85$, $p=0.36$) and no interaction between word type and exposure ($F(1,91)=0.04$, $p=0.85$)

A repeated measure Bayesian ANOVA demonstrated that a model that included only an effect of item type was the best performing model. When comparing all other models to the item type only model, results showed weak evidence against a model that added exposure (BF10=0.388), strong evidence against a model that added exposure and the interaction between item type and exposure (BF10=0.088) and extremely strong evidence against a model that included only exposure (BF10<0.01). Comparison of the different effects revealed extreme evidence for the inclusion of item type (BFIncl = 1011.4), weak evidence against inclusion of exposure (BFIncl = 0.317) and moderate evidence against inclusion of the interaction between item type and exposure (BFIncl = 0.254). Both methods of model comparison demonstrate evidence against an interaction between exposure and word type.
